# Fissureless technique of robotic left lingular segmentectomy for primary lung cancer with incomplete fissure: a case report

**DOI:** 10.1186/s13019-023-02211-7

**Published:** 2023-04-11

**Authors:** Masahiro Yanagiya, Masaaki Nagano, Jun Nakajima

**Affiliations:** Department of Thoracic Surgery, The University of Tokyo Graduate School of Medicine, 7-3-1 Hongo, Bunkyo-ku, Tokyo, 113-8655 Japan

**Keywords:** Thoracic surgery, Lung cancer, Segmentectomy, Robotic thoracic surgery, Robotic surgery

## Abstract

**Background:**

Pulmonary segmentectomy for a lung with an incomplete interlobar fissure may complicate persistent air leakage. The fissureless technique is often used in lobectomy to prevent persistent air leakage. We herein describe successful use of the fissureless technique for segmentectomy with the aid of a robotic surgical system.

**Case presentation:**

A 63-year-old man was clinically diagnosed with early-stage lung cancer for which lingular segmentectomy was indicated. A preoperative image revealed a lung with an incomplete fissure. Based on three-dimensional reconstruction imaging, we planned to divide the hilum structures in the order of the pulmonary vein, bronchus, and pulmonary artery and finally resect the lung parenchyma by dividing the intersegmental plane and interlobar fissure. This fissureless technique was successfully conducted using a robotic surgical system. The patient did not develop persistent air leakage and was alive without recurrence 1 year after segmentectomy.

**Conclusions:**

The fissureless technique may be a useful option in segmentectomy for a lung with an incomplete interlobar fissure.

**Supplementary Information:**

The online version contains supplementary material available at 10.1186/s13019-023-02211-7.

## Background

Segmentectomy may be a feasible option for surgical treatment of early-stage non-small cell lung cancer despite the fact that lobectomy with lymph node dissection has long been the standard treatment for primary lung cancer [1]. A recent multi-institutional prospective trial suggested that segmentectomy is superior to lobectomy in terms of overall survival for patients with early-stage non-small cell lung cancer [2].

Although segmentectomy is beneficial in terms of overall survival and preservation of lung parenchyma, persistent air leakage has been observed more frequently after segmentectomy than after lobectomy [3]. Persistent air leakage after anatomical resection may occur especially in patients with dense fissures [4, 5]. In these patients, the manipulation of interlobar fissures during segmentectomy complicates persistent air leakage.

The current report describes a fissureless technique for left upper lingular segmentectomy of early-stage primary lung cancer with a dense fissure. Although the fissureless technique has been proven effective in lobectomy for decreasing the incidence of persistent air leakage [6], we applied this technique to segmentectomy. Using a robotic system, we successfully performed fissureless lingular segmentectomy for primary lung cancer in a patient with a densely fused incomplete interlobar fissure.

## Case presentation

**Fig. 1:**
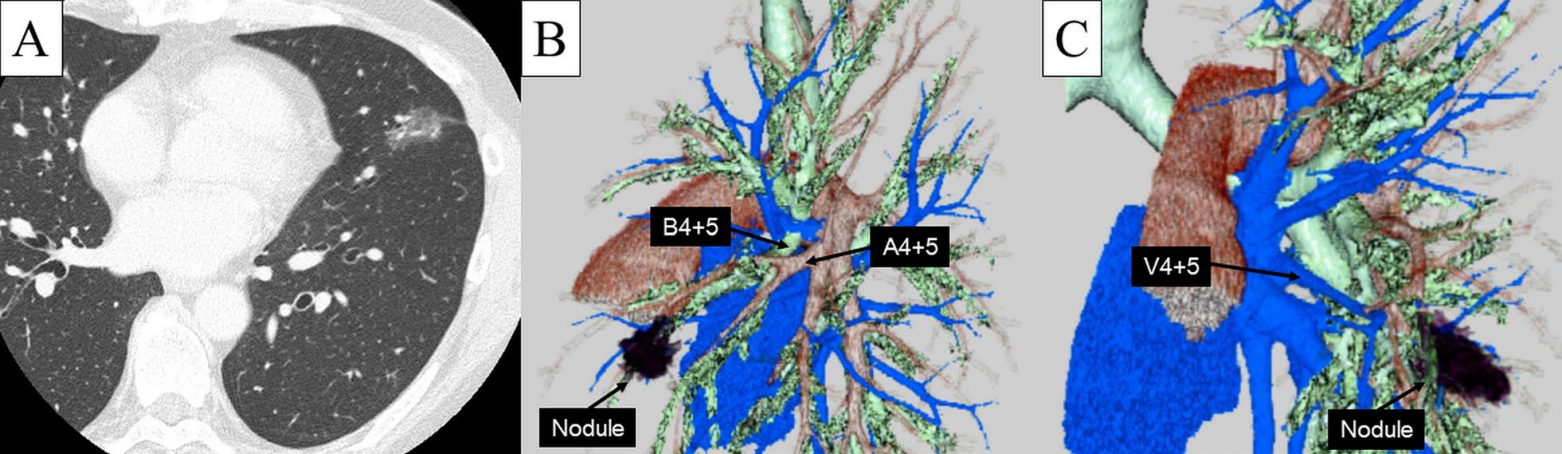
Preoperative chest computed tomography image and three-dimensional images. **A** Chest computed tomography showed a part-solid ground-glass nodule containing a solid component that was highly suspicious for malignancy. **B** The lingular pulmonary artery (A4 + 5) completely branched from the interlobar pulmonary artery. **C** The lingular pulmonary vein (V4 + 5) drained into the common trunk of the left pulmonary vein

A 63-year-old asymptomatic man with a history of ulcerative colitis was referred to our hospital because of an abnormal ground-glass lung nodule. The ground-glass nodule had been detected by chest computed tomography (CT). During 2 years of close follow-up, the nodule had gradually increased in size. CT finally showed a part-solid ground-glass nodule measuring 19 mm (the solid component measured 8 mm) in the left lingular segment, which raised suspicion for malignancy (Fig. 1A). 18F-fluorodeoxyglucose positron emission tomography (FDG-PET) showed hypometabolic activity (maximum standardized uptake value, 2.1). Distant metastases were not detected by whole-body CT or FDG-PET. The patient was suspected to have early-stage lung cancer (cT1aN0M0-IA1). Surgical resection was indicated.

Considering the tumor size and location, we decided to perform left lingular segmentectomy and lymph node dissection with the aid of a robotic system. The preoperative CT image suggested an incomplete interlobar fissure (Fig. 1A). The three-dimensional CT image was reconstructed by SYNAPSE VINCENT software (Fujifilm Medical, Tokyo, Japan). The lingular pulmonary artery (A4 + 5) completely branched from the interlobar pulmonary artery (Fig. 1B). The patient had a common trunk of the pulmonary vein (Fig. 1C). We adopted a fissureless segmentectomy technique to minimize persistent air leakage. We planned to sequentially dissect the pulmonary vein, segmental bronchus, and pulmonary artery from the anterior hilum. After division of the vascular and bronchial structures, we planned to finally divide the interlobar fissure and intersegmental plane with mechanical staplers.

**Fig. 2:**
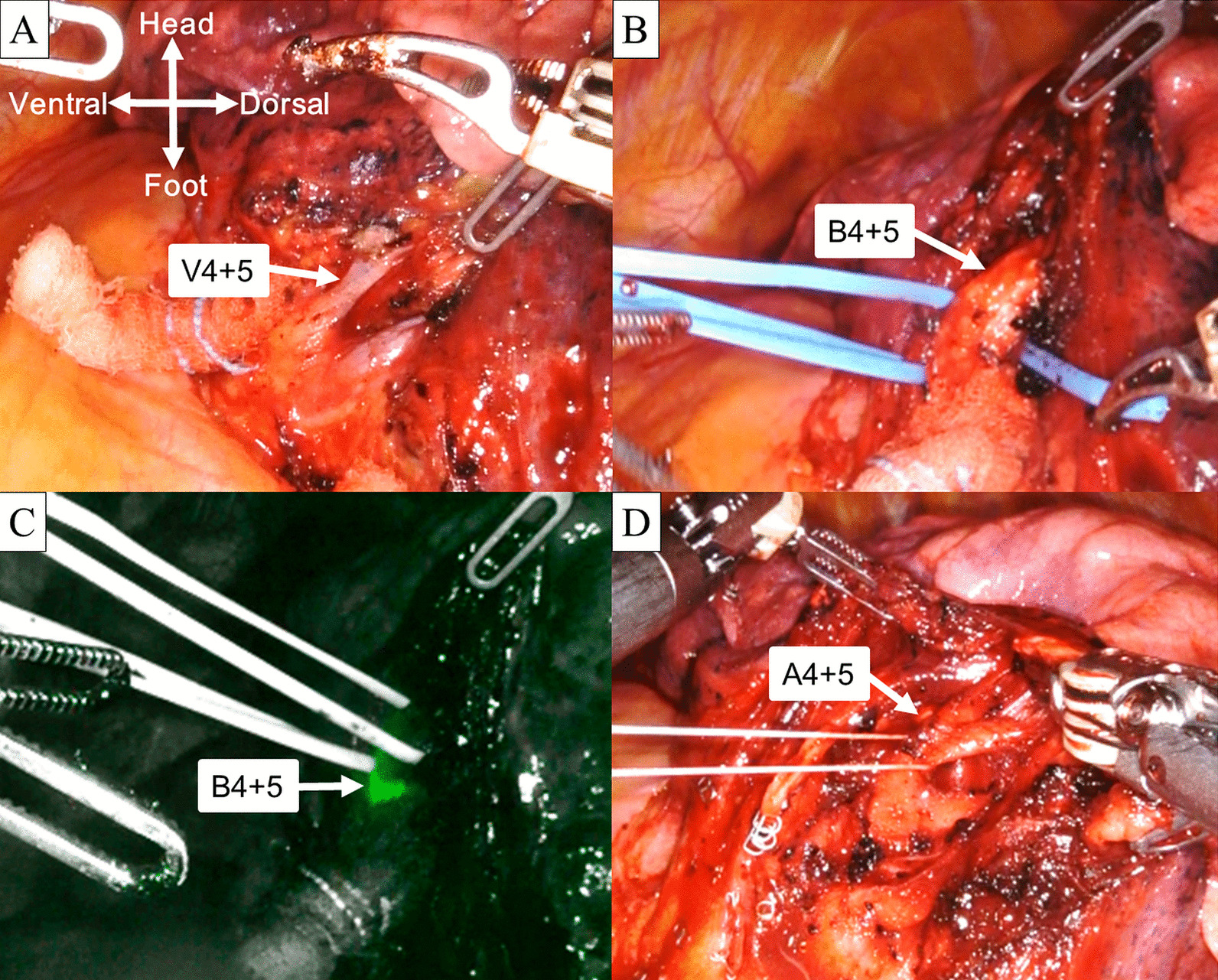
Intraoperative photographs of surgical field. **A** The lingular pulmonary vein (V4 + 5) was exposed from the hilum and identified. **B** The lingular bronchus (B4 + 5) was located immediately behind the dissected lingular pulmonary vein (V4 + 5). **C** Under Firefly mode, the light of the bronchoscope turned green, indicating the lingular bronchus (B4 + 5). **D** The lingular pulmonary artery (A4 + 5) was exposed immediately behind the dissected lingular bronchus (B4 + 5)

The surgery was conducted via four incisions and one assist incision. Four robotic ports were placed in the eighth intercostal space. The assist port was placed in the fifth intercostal space on the anterior axillary line. Prior to docking of the robotic system, we initially confirmed no pleural effusion, pleural dissemination, or adhesion under thoracoscopy. We also identified the tumor location using finger palpation. The da Vinci Xi Surgical System (Intuitive Surgical, Sunnyvale, CA, USA) was docked. We dissected the anterior hilum and exposed the pulmonary veins. Although this patient had a common trunk of the pulmonary vein, we were able to identify V4 + 5 based on the tumor location (Fig. 2A). V4 + 5 was exposed. V3 was partially exposed and taped to confirm both V4 + 5 and B4 + 5. V4 + 5 was dissected by a robotic stapler (Additional file 1). Because we knew that B4 + 5 branched immediately behind V4 + 5 based on the preoperative CT image, we exposed and taped B4 + 5 (Fig. 2B, Additional file 2). To confirm that the taped segmental bronchus was indeed B4 + 5, the console surgeon performed bronchoscopy and correctly identified B4 + 5 (Additional file 2). In addition to endobronchial confirmation of B4 + 5, we were also able to recognize B4 + 5 from the surgical field with the aid of the Firefly fluorescence image mode (Intuitive Surgical) (Fig. 2C). Under the Firefly mode, the light at the tip of the bronchoscope turned green (Fig. 2C, Additional file 2). B4 + 5 was dissected by a robotic stapler (Additional file 2). We then carefully exposed A4 + 5 branching from the interlobar pulmonary artery (Fig. 2D). A4 + 5 was also transected by a robotic stapler (Additional file 3).

**Fig. 3:**
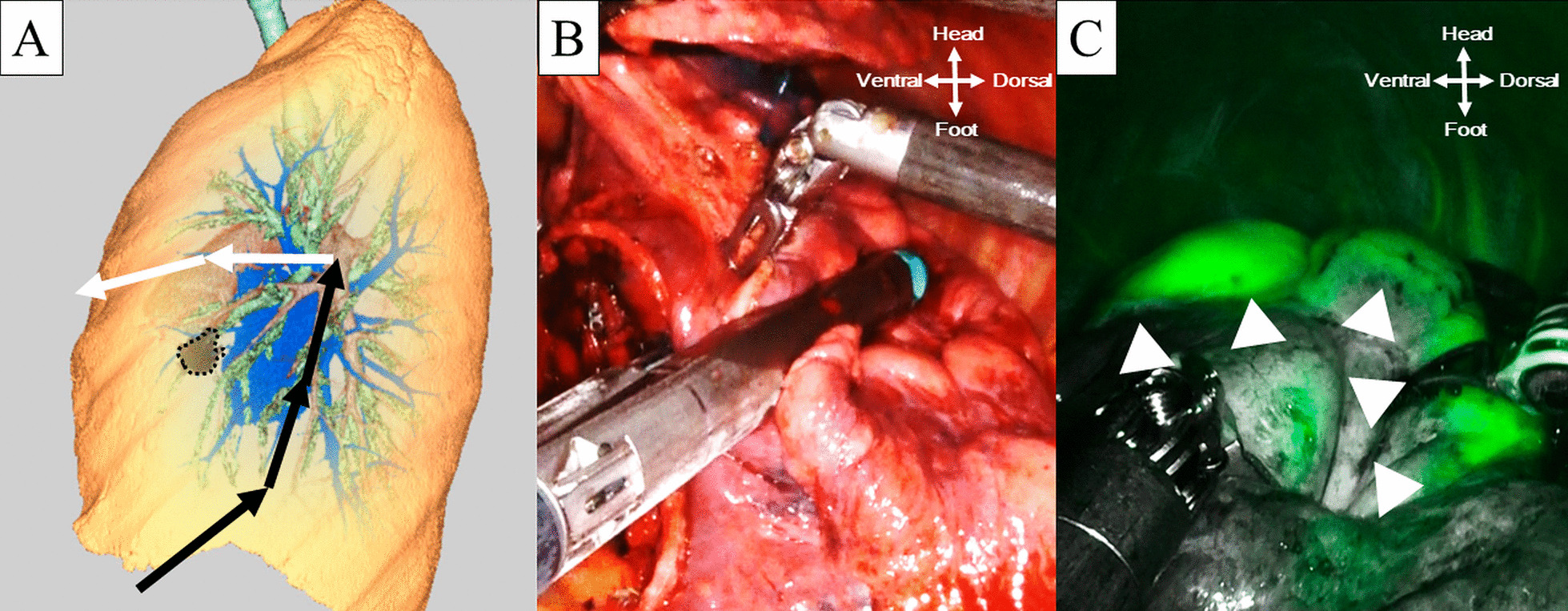
Division of interlobar fissure and intersegmental plane. **A** Image of division of lung parenchyma. The black lines indicate the resection lines considering the surgical margin around the interlobar fissure. The white lines indicate the intersegmental plane between the left upper division segment and lingular segment. The black dotted area indicates the tumor. **B** Division of lung parenchyma around the interlobar fissure. Concomitant resection of the anterior segment of the left lower lobe was conducted to obtain a surgical margin. **C** Delineation of the intersegmental plane by intravenous injection of indocyanine green under Firefly mode. The white arrows indicate the intersegmental plane

The resection line was designed to obtain a surgical margin (Fig. 3A). In the area around the tumor, we planned to concomitantly resect part of the anterior segment of the lower lobe (S8) to secure a surgical margin because the tumor was near the interlobar fissure (Fig. 3B). After division of part of the interlobar fissure, 4 mL (10 mg) of indocyanine green (ICG) was injected intravenously to identify the interlobar fissure and intersegmental plane. The surgical field was visualized with the Firefly fluorescence image mode (Fig. 3C, Additional file 4). The interlobar fissure line and intersegmental line were identified as the border separating the green lung parenchyma (remaining parenchyma) from the dark lung parenchyma (resected parenchyma) (Fig. 3C). The lung parenchyma was divided by mechanical staplers as planned (Additional file 4). The main branch of V3 was preserved. We achieved extended lingular segmentectomy using the fissureless approach. We obtained a surgical margin of more than 2 cm. The frozen section diagnosis of the resected specimen was adenocarcinoma. We subsequently conducted systematic lymph node dissection. The whole surgical time was 218 min, and the console time was 134 min. Only slight blood loss occurred. The chest tube was removed on postoperative day 3, and the patient was discharged on postoperative day 7.

The patient developed no complications such as persistent air leakage. The final pathological diagnosis was invasive adenocarcinoma with an invasion size of 8 mm and total size of 15 mm. The pathological stage was p-T1aN0M0. The patient was alive without recurrence 1 year postoperatively.

## Discussion

We have herein described the successful performance of a fissureless technique of left lingular segmentectomy for lung cancer in a patient with an incomplete fissure. Because a recent prospective trial demonstrated promising outcomes of segmentectomy [2], thoracic surgeons are more likely to manage challenging cases as shown in the current case. This case report offers an optional technique to manage an incomplete fissure during segmentectomy. In an assessment of 250 patients by high-resolution chest CT, a left incomplete fissure was found in 24% [7]. Thus, this technique might be required in approximately one-quarter of all lingular segmentectomies. The three key points for successful performance of this surgery are preoperative CT image reconstruction, intraoperative tumor identification, and systemic ICG injection.

Preoperative three-dimensional CT image reconstruction is necessary to successfully complete this surgery. Because the segmental anatomy is highly various [8–12], surgeons must understand the patient’s detailed anatomy. In our patient, A4 + 5 branched from the interlobar pulmonary artery, not from the mediastinal pulmonary artery. Thus, we were easily able to plan the procedure to cut the vessels and the bronchus.

Tumor localization is also important when performing this surgery. Our patient had a common trunk, which reportedly exists with an incidence of 6.5–17.0% [11, 13, 14]. V4 + 5 is usually difficult to identify in the presence of a common trunk [12]. In our patient, however, the location of the tumor made it easier to identify and confirm V4 + 5. Tumor localization greatly helps to understand the anatomy and safely perform the surgery. In addition, tumor identification is important in terms of securing the surgical margin [15]. Although segmentectomy is promising in terms of overall survival, the procedure may have a higher risk of local recurrence [2]. A secure surgical margin is required to achieve curative segmentectomy and thus prevent local recurrence [15]. In the present case, we obtained a sufficient surgical margin because of successful tumor identification. Although it was easy to localize the tumor in the current case, a tumor localization technique such as virtual-assisted lung mapping might be necessary for an impalpable tumor appearing as a pure ground-glass nodule located deep to the pulmonary surface [16].

Systemic ICG injection and the fluorescence image mode were highly valuable during the surgery in this case. Previous reports have shown the efficacy of systemic ICG injection in terms of identification of intersegmental planes [17]. Systemic ICG injection has been widely applied in segmentectomy [18]. In the current case, intraoperative ICG injection facilitated identification of not only the intersegmental plane but also the interlobar fissure [19]. The present case shows that systemic ICG injection is useful for challenging cases involving a patient with an incomplete fissure.

In addition to these three key points, the robotic surgical system greatly contributed to this surgery. Because of the operability of the robotic surgical system, the console surgeon was able to easily and meticulously dissect the vessels and bronchus [20]. Moreover, the console surgeon was able to perform bronchoscopy to precisely confirm the anatomy. Previous reports have suggested that segmentectomy might require skilled bronchoscopists other than surgeons to confirm the bronchus and intersegmental plane [18]. One of the advantages of performing segmentectomy by robotic surgery is that a skilled bronchoscopist is not required.

Although the fissureless technique for lobectomy has been widely demonstrated in the literature [4–6], reports of the fissureless technique for segmentectomy are limited [21]. One report described two cases of fissureless segmentectomy via a four-port thoracoscopic approach using an inflation–deflation technique [21]. The authors showed promising results in terms of the efficacy and safety of this fissureless technique [21]. Unlike their approach, we conducted fissureless segmentectomy via a robotic approach using systemic ICG injection.

## Conclusion

We successfully conducted robotic left lingular segmentectomy via the fissureless technique. This technique is an optional surgical procedure for a lung with an incomplete fissure.

## Supplementary Information

**Additional file 1.** Dissection of pulmonary vein

**Additional file 2.** Dissection of bronchus

**Additional file 3.** Dissection of pulmonary artery

**Additional file 4.** Division of lung parenchyma

## Data Availability

All data generated or analyzed are included in this article.

## Abbreviations

CT: Computed tomography
FDG-PET: 18F-fluorodeoxyglucose positron emission tomography
ICG: Indocyanine green

## References

[1] Okada M, Koike T, Higashiyama M, Yamato Y, Kodama K, Tsubota N (2006). Radical sublobar resection for small-sized non-small cell lung cancer: a multicenter study. J Thorac Cardiovasc Surg.

[2] Saji H, Okada M, Tsuboi M, Nakajima R, Suzuki K, Aokage K (2022). Segmentectomy versus lobectomy in small-sized peripheral non-small-cell lung cancer (JCOG0802/WJOG4607L): a multicentre, open-label, phase 3, randomised, controlled, non-inferiority trial. Lancet.

[3] Suzuki K, Saji H, Aokage K, Watanabe SI, Okada M, Mizusawa J (2019). Comparison of pulmonary segmentectomy and lobectomy: Safety results of a randomized trial. J Thorac Cardiovasc Surg.

[4] Stamenovic D, Bostanci K, Messerschmidt A, Jahn T, Schneider T (2016). Fissureless fissure-last video-assisted thoracoscopic lobectomy for all lung lobes: A better alternative to decrease the incidence of prolonged air leak?. Eur J Cardiothorac Surg.

[5] Igai H, Kamiyoshihara M, Yoshikawa R, Osawa F, Kawatani N, Ibe T (2016). The efficacy of thoracoscopic fissureless lobectomy in patients with dense fissures. J Thorac Dis.

[6] Li SJ, Zhou K, Li YJ, Li PF, Wu YM, Liu LX (2017). Efficacy of the fissureless technique on decreasing the incidence of prolonged air leak after pulmonary lobectomy: a systematic review and meta-analysis. Int J Surg.

[7] Heřmanová Z, Ctvrtlík F, Heřman M (2014). Incomplete and accessory fissures of the lung evaluated by high-resolution computed tomography. Eur J Radiol.

[8] Isaka T, Mitsuboshi S, Maeda H, Kikkawa T, Oyama K, Murasugi M (2020). Anatomical analysis of the left upper lobe of lung on three-dimensional images with focusing the branching pattern of the subsegmental veins. J Cardiothorac Surg.

[9] Shimizu K, Nagashima T, Ohtaki Y, Obayashi K, Nakazawa S, Kamiyoshihara M (2016). Analysis of the variation pattern in right upper pulmonary veins and establishment of simplified vein models for anatomical segmentectomy. Gen Thorac Cardiovasc Surg.

[10] Zhang M, Mao N, Zhang K, Zhang M, Liu Y, Wang RF (2020). Analysis of the variation pattern in left upper division veins and establishment of simplified vein models for anatomical segmentectomy. Ann Transl Med.

[11] Marom EM, Herndon JE, Kim YH, McAdams HP (2004). Variations in pulmonary venous drainage. Variations in pulmonary venous drainage to the left atrium: implication for radiofrequency ablation. Radiology.

[12] Endo T, Tetsuka K, Yamamoto S, Aizawa K, Endo S (2012). Transection of left common pulmonary vein during left upper lobectomy: how should it be reconstructed?. J Surg Case Rep.

[13] Mansour M, Holmvang G, Sosnovik D, Migrino R, Abbara S, Ruskin J (2004). Assessment of pulmonary vein anatomic variability by magnetic resonance imaging: implications for catheter ablation techniques for atrial fibrillation. J Cardiovasc Electrophysiol.

[14] Cronin P, Kelly AM, Desjardins B, Patel S, Gross BH, Kazerooni EA (2007). Normative analysis of pulmonary vein drainage patterns on multidetector CT with measurements of pulmonary vein ostial diameter and distance to first bifurcation. Acad Radiol.

[15] Sato M (2020). Precise sublobar lung resection for small pulmonary nodules: localization and beyond. Gen Thorac Cardiovasc Surg.

[16] Sato M, Kobayashi M, Kojima F, Tanaka F, Yanagiya M, Kosaka S (2018). Effect of virtual-assisted lung mapping in acquisition of surgical margins in sublobar lung resection. J Thorac Cardiovasc Surg.

[17] Misaki N, Chang SS, Igai H, Tarumi S, Gotoh M, Yokomise H (2010). New clinically applicable method for visualizing adjacent lung segments using an infrared thoracoscopy system. J Thorac Cardiovasc Surg.

[18] Andolfi M, Potenza R, Seguin-Givelet A, Gossot D (2020). Identification of the intersegmental plane during thoracoscopic segmentectomy: state of the art. Interact Cardiovasc Thorac Surg.

[19] Okazaki M, Suzawa K, Shien K, Miyoshi K, Otani S, Yamamoto H, Sugimoto S, Yamane M, Toyooka S (2021). Robot-assisted thoracoscopic lobectomy for severe incomplete interlober fissure. J Surg Case Rep.

[20] Abbas AE (2017). Robotic portal lobectomy, surgery through a virtual thoracotomy. J Thorac Dis.

[21] Akiba T, Nakada T, Inagaki T (2015). Simulation of the fissureless technique for thoracoscopic segmentectomy using rapid prototyping. Ann Thorac Cardiovasc Surg.

